# Cul o 2 specific IgG3/5 antibodies predicted *Culicoides* hypersensitivity in a group imported Icelandic horses

**DOI:** 10.1186/s12917-020-02499-w

**Published:** 2020-08-10

**Authors:** Fahad Raza, Renata Ivanek, Heather Freer, Dania Reiche, Horst Rose, Sigurbjörg Torsteinsdóttir, Vilhjálmur Svansson, Sigríður Björnsdóttir, Bettina Wagner

**Affiliations:** 1 Departments of Population Medicine and Diagnostic Sciences, College of Veterinary Medicine, Cornell University, Ithaca, NY 14853 USA; 2 Boehringer Ingelheim Vetmedica, 55216 Ingelheim, Germany; 3 Institute for Experimental Pathology, University of Iceland, Keldnavegur 3, 112 Reykjavik, Keldur, Iceland; 4 Icelandic Food and Veterinary Authority, MAST, Office of Animal Health and Welfare, 800 Selfoss, Iceland

**Keywords:** *Culicoides* hypersensitivity, Major allergens, Horse, Allergy, IgG, IgE, Clinical score, Immunologically naïve, Biomarkers

## Abstract

**Background:**

*Culicoides* hypersensitivity (CH) is induced in horses by salivary allergens of *Culicoides* midges. In Iceland, the causal *Culicoides* species for CH are not present. Previous epidemiological data indicated that Icelandic horses are more susceptible to CH when they are exported from Iceland and first exposed to *Culicoides* at adult age. Horses born in countries where *Culicoides* is endemic, develop the disease less frequently. Here, we established a longitudinal allergy model to identify predictive and diagnostic serological biomarkers of CH.

**Results:**

Sixteen adult Icelandic horses from Iceland were imported to the Northeastern United States (US) during the winter and were kept in the same environment with natural *Culicoides* exposure for the next two years. None of the horses showed clinical allergy during the first summer of *Culicoides* exposure. In the second summer, 9/16 horses (56%) developed CH. Allergen specific IgE and IgG isotype responses in serum samples were analysed using nine potential *Culicoides* allergens in a fluorescent bead-based multiplex assay. During the first summer of *Culicoides* exposure, while all horses were still clinically healthy, Cul o 2 specific IgG3/5 antibodies were higher in horses that developed the allergic disease in the second summer compared to those that did not become allergic (*p* = 0.043). The difference in Cul o 2 specific IgG3/5 antibodies between the two groups continued to be detectable through fall (*p* = 0.035) and winter of the first year. During the second summer, clinical signs first appeared and Cul o 3 specific IgG3/5 isotypes were elevated in allergic horses (*p* = 0.041). Cul o 2 specific IgG5 (p = 0.035), and Cul o 3 specific IgG3/5 (p = 0.043) were increased in late fall of year two when clinical signs started to improve again.

**Conclusions:**

Our results identified IgG5 and IgG3/5 antibodies against Cul o 2 and Cul o 3, respectively, as markers for CH during and shortly after the allergy season in the Northeastern US. In addition, Cul o 2 specific IgG3/5 antibodies may be valuable as a predictive biomarker of CH in horses that have been exposed to *Culicoides* but did not yet develop clinical signs.

## Background

*Culicoides* hypersensitivity (CH) is an allergic disease in adult horses known by several names, such as summer eczema, sweet itch, summer seasonal recurrent dermatitis, insect bite hypersensitivity, and others [1, 2]. CH is an immunoglobulin E (IgE) mediated type-I hypersensitivity caused by bites of *Culicoides* midges [3–5]. Affected horses develop a seasonal recurrent allergic dermatitis. Clinical signs start in spring or early summer while *Culicoides* are present in the environment and include pruritus, loss of hair, skin irritation, and open wounds. The initially acute dermatitis develops into chronic skin lesions during the summer and as long as the horses are continuously exposed to *Culicoides* in their environment [3, 5–7]. Skin lesions typically occur at the preferred feeding sites of *Culicoides*, can differ by geographic location and species of the midges, and can generally include the ventral midline, head, chest, neck, mane, and tail, and other skin areas [7]. Due to the extreme discomfort of CH, severely affected horses kept on pasture experience weight loss during the summer while their herd mates gain weight. Clinical signs start to improve in fall with decreasing *Culicoides* exposure and can solve completely during the winter. Although CH is not a life-threatening disease, it massively affects the well-being and performance of the affected horses for an extended time during the summer [2, 8].

CH affects all breeds of horses although the prevalence is highly variable (4–70%) [9–13]. The risk of developing the allergic condition is particularly high for adult Icelandic horses born in Iceland after export to countries where *Culicoides* is endemic [14, 15]. *Culicoides* species feeding on horses have not been found in Iceland [5, 7, 16]. Exported adult Icelandic horses often develop clinical allergy during their second summer of exposure to *Culicoides* midges [5, 7].

The allergic skin reaction can be transferred to healthy horses by intradermal (i.d.) injection of IgE from allergic individuals followed by i.d. injection of *Culicoides* extract [3]. In addition to IgE, the involvement of IgG3/5 in Fc-receptor-mediated degranulation of equine mast cells has been discussed [3, 4, 6, 7]. In particular, one monoclonal antibody (mAb) against equine IgG3/5, clone CVS40, provoked immediate skin reaction after i.d. injection [3, 4]. However, i.d. injection of several other mAbs against IgG3/5, IgG5 or IgG1/3 did not induce any skin reaction [4].

Salivary proteins of *Culicoides* midges can cause the allergic reaction by cross-linking allergen specific IgE on the surface of skin mast cells in affected horses [3, 4, 17, 18]. Several salivary proteins from different *Culicoides* species, such as *C. nubeculosus, C. obsoletus*, and *C. sonorensis*, have been identified as potential allergens by various groups [6, 10, 19–22]. The classification of these proteins as major and minor allergens of CH still has to be confirmed for various region and environments around the world.

*Culicoides* midges are active from early summer to fall [5, 7]. During the winter months, when midges are absent from the environment, clinical allergy wanes and CH affected horses get a temporary relief from clinical signs until horses are exposed again to *Culicoides* [4, 5]. All current treatment options of CH are symptomatic, offering at best temporary relief but do not cure the allergic condition. Tests or biomarkers to identify horses that may develop CH at an early stage and prior to clinical allergy do not yet exist.

**Fig. 1:**
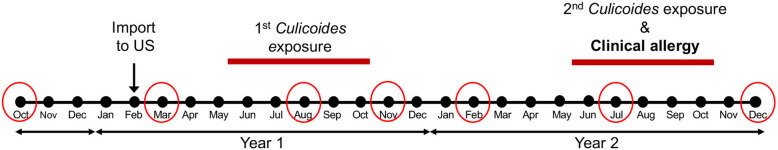
Experimental outline of the longitudinal *Culicoides* hypersensitivity study. Icelandic horses (*n* = 16) were exported from Iceland at adult age. Importation to the US took place in February of year 1 (arrow). The blood sampling time points (red circles) are shown for the two-year duration of the study. Bold horizontal bars indicate the months of exposure to *Culicoides* in the environment of the horses, typically from mid-May to mid-October. Clinical signs of allergy started during the second summer of *Culicoides* exposure

Here, we established a longitudinal CH model in horses that were exported from Iceland to the United States of America (US) during the winter and were kept for the following two years in a defined environment with natural exposure to *Culicoides*. Serum samples were obtained from the horses at several times to identify serum biomarkers as predictors and indicators of clinical allergy (Fig. 1). Clinical signs were recorded during the two-year study period and allowed the grouping of the horses into allergic and non-allergic individuals. Serum samples were analysed for allergen specific IgG isotypes and IgE using a new allergen multiplex assay with nine potential *Culicoides* salivary allergens. Allergen specific IgG and IgE data were then analysed by asking the following questions: 1) Can allergen specific IgG or IgE distinguish between allergic and non-allergic horses prior to the development of clinical signs of CH? 2) Are there any differences detectable between the two groups prior to the first *Culicoides* allergen exposure? 3) Which allergen specific IgG or IgE combinations can be used to differentiate between allergic and non-allergic horses while clinical signs of CH are present? and 4) Can allergen specific IgG or IgE isotypes differentiate between allergic and non-allergic groups after CH first occurred and while clinical signs were not apparent anymore (e.g. during the winter)?

## Results

### Development of clinical allergy

**Fig. 2:**
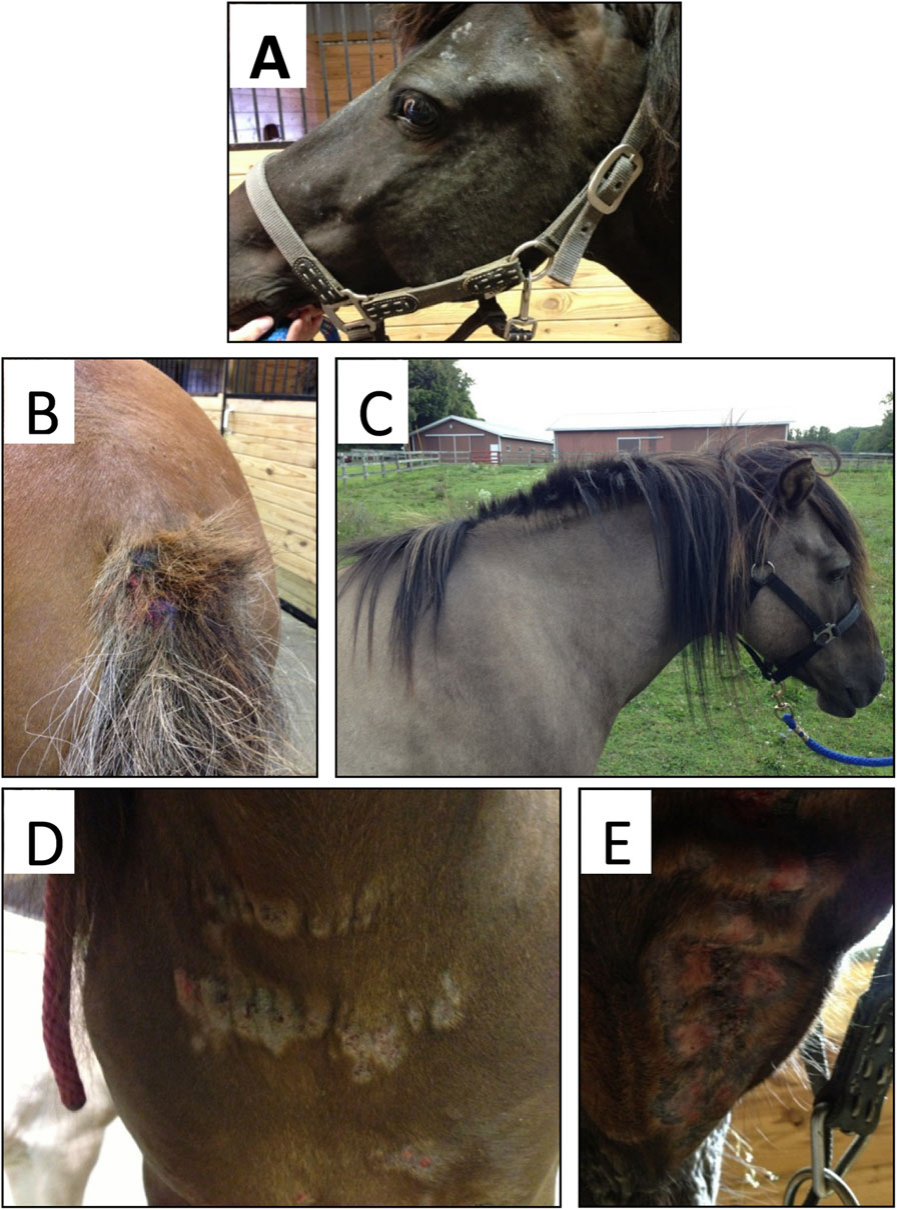
Clinical signs of *Culicoides* hypersensitivity. Allergic horses during the summer of year 2 are shown: (**a**) skin lesion on the face, (**b**) hair loss and wounds on the tail and (**c**) mane, (**d**) allergic wounds on the chest and (**e**) intermandibular space

All 16 *Culicoides* naïve horses included in this two-year study remained clinically healthy during the first summer of natural *Culicoides* exposure. Several horses started to occasionally rub their mane and/or tail and mild alopecia was observed at the face or chest resulting in a slight elevation of their clinical scores during their first summer in the US. During the second summer after import, the horses were again exposed to *Culicoides*. Seven horses remained clinically healthy showing the same slight increase in their clinical scores as in year 1, while nine horses (56%) developed clinical allergy including dermatitis (Fig. 2).

**Fig. 3:**
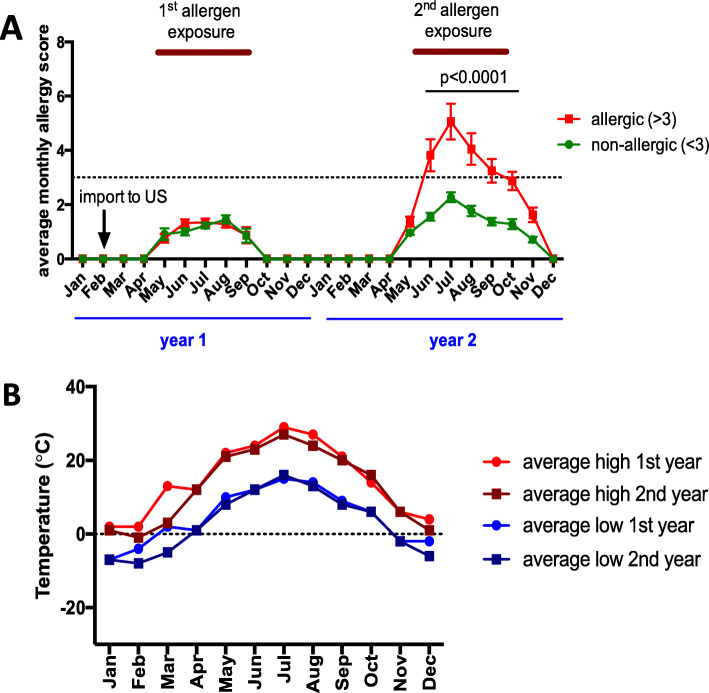
Clinical allergy scores in adult Icelandic horses during the first two years of natural exposure to *Culicoides* midges. **a**) Sixteen horses were exported from Iceland to the US in the winter (February) of year 1 and were all kept in the same environment with natural *Culicoides* exposure during the summer until the end of year 2. The severity of clinical allergy was evaluated by clinical allergy scoring. In the summer of year 2, nine horses became allergic while seven horses remained non-allergic. The graphs show means and standard errors of the monthly allergy scores. The dotted line shows the established cut-off value of the allergy scoring system. **b**) Comparison of the average monthly low and high temperatures in the environment of the horses during the first and second year of *Culicoides* exposure revealed similar temperature curves in both years

Clinical signs of allergy were observed between early June and mid-October of year 2 (all *p* < 0.0001), coinciding with the seasonal presence of *Culicoides* in the environment of the horses (Fig. 3a). Allergic horses were affected on the face, their intermandibular space, chest and/or ventral midline. Several horses showed loss of hair at the mane and/or tail (Fig. 2). After the first frost mid-October, clinical signs resolved and clinical scores in allergic horses became again comparable to the non-allergic group. However, the allergic episode during the summer was still visible in some horses for another 1–2 months depending on allergy severity of the individual horse. In allergic horses, mane and tail hair were growing back in fall while dry skin crusts were still found at the previous locations of dermatitis. The comparison of average monthly high and low environmental temperature between years 1 and 2 did not show differences between years, with the exception of slightly elevated temperatures in March of year 1 (Fig. 3b).

The group assignments into allergic and non-allergic horses were based on the occurrence of clinical signs of allergy in the second summer of *Culicoides* exposure. The group assignment was applied to the entire two-year observation period of this study to identify possible differences in allergen specific antibody responses between the two groups.

### Serological predictors of clinical allergy

**Fig. 4:**
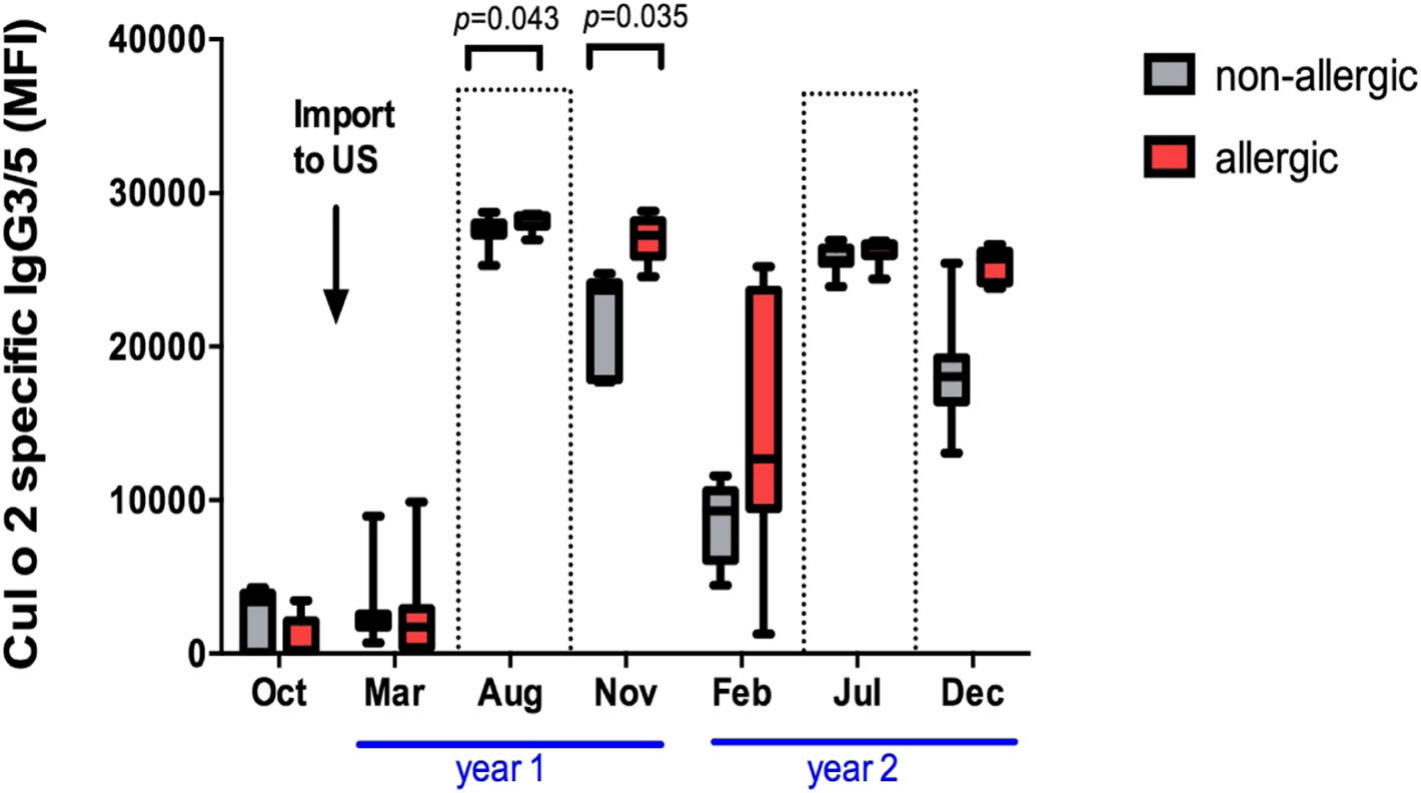
Cul o 2 specific IgG3/5 antibodies in serum of allergic and non-allergic horses. IgG3/5 antibodies in serum of allergic (*n* = 9) and non-allergic (*n* = 7) horses were measured using a *Culicoides* allergen multiplex assay. Horses were imported to the US in the beginning of year 1 (arrow). The areas surrounded by dotted lines represent the natural exposure times to *Culicoides* midges during the two-year study period. MFI = median fluorescence intensity

**Fig. 5:**
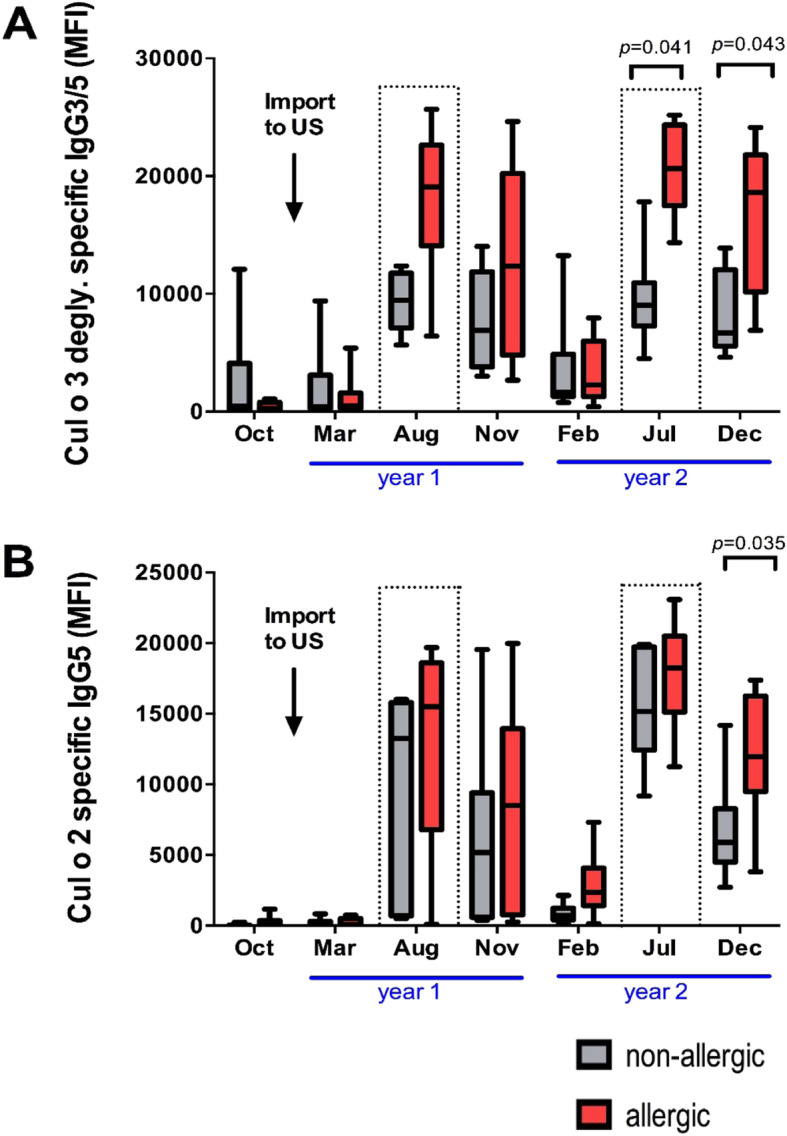
Allergen specific IgG3/5 and IgG5 responses against deglycosylated Cul o 3 and Cul o 2. IgG3/5 and IgG5 antibodies were measured using a *Culicoides* allergen multiplex assays. Longitudinal data representing (**a**) Cul o 3 specific IgG3/5 antibodies and (**b**) Cul o 2 specific IgG5 antibodies in serum of allergic (n = 9) and non-allergic (n = 7) horses during the two-year study period. The arrow shows the time of import of the horses to the US. The areas within the dotted lines indicate times of natural exposure to *Culicoides* midges. MFI = median fluorescence intensity

Allergen specific IgG isotype responses were measured in serum samples from the horses using a *Culicoides* allergen multiplex assay based on nine potential salivary allergens. Our longitudinal model allowed us to analyze if individual allergen specific isotypes distinguished allergic from non-allergic horse as potential predictive markers of CH before horses developed clinical allergy in year 2. Allergen specific antibodies in serum samples taken while the horses were still in Iceland (October prior to import) or in spring (March) of year 1 after import to the US but prior to *Culicoides* exposure were overall low and did not result in serological differences that could separate the two groups (Figs. 4 and 5).

During their first summer in the US all horses were exposed to *Culicoides* midges and responded with increased serum antibodies against *Culicoides* salivary allergens. Nevertheless, clinical signs of allergy were not observed. In August of year 1, Cul o 2 specific IgG3/5 serum antibodies increased significantly in the allergic group (*p* = 0.043) compared to non-allergic horses (Fig. 4). After the *Culicoides* exposure season in November of year 1, Cul o 2 specific IgG3/5 antibodies were still elevated in the allergic group (*p* = 0.035; Fig. 4). Afterwards, a trend of having overall higher Cul o 2 specific IgG3/5 antibodies was maintained in the allergic compared to the non-allergic group but did not reach significance anymore. This result supported the conclusion that elevated Cul o 2 specific IgG3/5 antibodies during or immediately after the first summer of exposure to *Culicoides* predicted the development of CH in these horses in the following summer.

None of the other allergen specific Ig isotypes were serologically different between the allergic and non-allergic groups between the summer of year 1 and February of year 2. Cul o 2 specific IgG1 and IgG1/3 antibodies were slightly elevated in the allergic group in August of year 1 but did not reach significance. The Cul o 2 specific IgG1, IgG1/3, IgG4/7, IgG6 and IgE responses are shown in Additional file 2.

### Allergen specific serum IgG responses indicative of clinical allergy

The *Culicoides* allergen multiplex assay was used to identify allergen specific Ig isotype that serologically distinguished allergic and non-allergic horses when clinical allergy developed during the second *Culicoides* exposure in the summer of year 2. In July of year 2, allergic horses had higher serum IgG3/5 antibodies against the Cul o 3 degly allergen (*p* = 0.041) compared to non-allergic horses (Fig. 5a). All of the remaining IgG isotype-allergen combinations showed no difference between the groups during this time point. The Cul o 3 degly specific IgG1, IgG1/3, IgG4/7, IgG5, IgG6 and IgE responses are shown in Additional file 3.

In the late fall of year 2, clinical signs of allergy resolved. Despite the absence of clinical signs in December of year 2, allergic horses still maintained higher serum IgG3/5 antibodies against Cul o 3 degly allergen than non-allergic horses (*p* = 0.043) (Fig. 5a). In addition, a significant increase in serum IgG5 values against Cul o 2 (*p* = 0.035) was also observed in allergic horses in December of year 2 after clinical signs resolved (Fig. 5b).

### Allergen specific IgE responses

We also used the allergen multiplex assay to detect allergen specific IgE in serum. Overall, the allergen specific IgE signals in serum obtained with this assay were low throughout the study period compared to the much higher IgG antibody responses. Differences in allergen specific IgE responses between allergic and non-allergic horses were not observed in these horses during the first two years of *Culicoides* exposure. Cul o2 and Cul o 3 degly specific IgE responses are shown in Additional files 2 and 3.

## Discussion

*Culicoides* midges feeding on horses are not present in Iceland and, consequently, horses in Iceland are immunologically naïve to *Culicoides* allergens [5, 7, 16]. Here, we studied the development of clinical signs of CH together with serological responses to nine *Culicoides* allergens in sixteen Icelandic horses that were imported to the US in February 2012 and kept in a *Culicoides* endemic environment for the next two years with natural exposure to the midges during the summer. Clinical signs of allergy were evaluated using a clinical scoring system [23] and horses were grouped in allergic and non-allergic groups after they developed clinical allergy or not in the second summer of *Culicoides* exposure. *Culicoides* allergen specific antibodies in serum were analyzed using a newly developed allergen multiplex assay before, during, and after horses showed clinical signs of CH for the first time.

In previous studies, serological responses were evaluated after horses showed clinical allergy in serum samples typically collected during *Culicoides* exposure [6, 7, 24–27]. In contrast, our approach used a uniquely controlled horse CH model and also analyzed allergen specific Ig isotypes in serum of pre-symptomatic horses and during seasons with and without *Culicoides* exposure. To the best of our knowledge, this is the first study looking prospectively at serological responses during allergy development in CH.

In our study group, clinical signs of CH developed in 56% of the horses in the second summer of *Culicoides* exposure which was similar to previous studies on exported horses [5, 7]. The absence of clinical signs of allergy in the first summer was not unexpected because IgE-mediated immune responses often need some time to develop and mast cell sensitization needs to occur prior to the development of clinical allergy [3, 4]. Several reports confirmed sensitization to *Culicoides* allergens by intradermal testing and showed that sensitization often occurs during the first season of *Culicoides* exposure with many of the sensitized horses remaining non-allergic in the following years [4, 5, 28–30]. Consequently, intradermal testing and sensitization are not reliable predictors whether a horse will develop allergy in the future or not. Thus, the identification of an allergic horse prior to the onset of clinical allergy is still a challenge.

One important finding in our prospective study was the identification of a potential predictive biomarker of CH after initial sensitization to *Culicoides* allergens but before signs of clinical allergy developed. Horses that developed clinical allergy, had higher Cul o 2 specific IgG3/5 serum antibody amounts than the non-allergic group in summer and fall of the first year in the US and this trend continued through the following winter. Cul o 2 specific IgG3/5 was detected in these horses several months before onset of clinical allergy and as early as three months following first exposure to *Culicoides* allergens.

A reliable serological marker, that allows the identification of horses at risk of becoming allergic while they are still clinically healthy, will open the door for the development of novel targeted preventive and therapeutic strategies that can interfere earlier and more effectively with the immune imbalance against *Culicoides* allergens. Currently, a causal treatment for CH is missing. Available treatments are given at a late stage after sensitization occurred, the immune response to the allergen clearly dysregulated, and becomes obvious as clinical disease [5, 9]. Thus far, a number of attempts were made to desensitize horses with allergen specific immunotherapy with low or inconsistent success [31–36]. Allergen immunotherapy in humans and studies in mouse models highlighted the importance of the early disease stage for successful therapeutic intervention and the development of allergen tolerance [37–41]. A predictive biomarker for CH would be advantageous for transforming therapeutic interventions and the search for a cure to this common allergic dermatitis of horses.

Nevertheless, the identification of Cul o 2 specific IgG3/5 was performed in a small number of horses living in one environment and under controlled conditions in respect to their nutrition, vaccination, deworming and other possible confounders of CH. Thus, this study is a proof-of-principle approach showing that predictive biomarkers can likely be detected for CH using modern sensitive technologies with allergen and isotype specific approaches. Thus, Cul o 2 specific IgG3/5 as a predictive biomarker requires further evaluation by using larger numbers of horses living in different environmental and housing conditions before it could be used as a more universal predictive marker for CH. We clearly observed some overlap in Cul o 2 specific IgG3/5 antibody amounts in allergic and non-allergic groups. This likely means that a future validated predictive biomarker assay for CH may need to have a high cut-off value to exclude all future healthy horses. Consequently, such an assay will not identify all horses that will develop future allergy. However, the results shown here indicate that the early identification of high values of Cul o 2 specific IgG3/5 could have value as a biomarker in a validated biomarker assay to identify future allergic horses above the assay’s cut-off value and range of serological overlap. These high-risk horses could become the target population for preventive and early allergen immunotherapy.

Currently, the major *Culicoides* allergens causing CH in the Northeastern US are unknown. Horses are exposed to many *Culicoides* salivary proteins, but only a few of these proteins will cause the degree of mast cell degranulation that leads to CH. Therefore, identification of disease relevant allergens will allow more specific diagnosis and treatment in the future. Out of nine potential *Culicoides* allergens tested in our approach, only two, Cul o 2 and Cul o 3 degly, resulted in significant differences in serological responses between the allergic and non-allergic groups. Cul o 3 responses showed a similar pattern as those to Cul o 3 degly (data not shown). Previous studies in allergic horses in different countries in Europe also identified Cul o 3 and Cul o 2 as major *Culicoides* allergens along with Cul s 1, Cul n 4 and Cul n 8 [6, 7, 42, 43]. Our data suggest that Cul o 2 and Cul o 3 also provide relevant diagnostic and possibly therapeutic allergens against CH in the Northeastern US. Although *C. obsoletus* has been described in Europe and not in the US, *C. sonorensis* is prevalent in the Northeast US [6]. It is thus likely that the immunogenic structures of Cul o 2 and Cul o 3 equivalents of *Culicoides* midges at our location are shared by different *Culicoides* species including those prevalent in Europe. In conclusion, both Cul o 2 and Cul o 3 have been reported as major allergens of *Culicoides* and are likely causing CH in different countries and continents.

In this study, we did not detect any serological difference of the latter allergic and non-allergic horses before they were imported to the US. This was an expected finding and coincides with previous studies showing Icelandic horses not reacting to intradermal *Culicoides* allergen injections while in Iceland [7, 16]. However, Ziegler et al. [7] found higher serum Cul o 3 specific IgG5 levels prior to their first exposure to *Culicoides* in horses from Iceland that were imported to Europe and later became allergic. It remains to be elucidated how Cul o 3 specific antibodies can develop in the absence of *Culicoides* exposure. In our study, both groups of horses maintained similar Cul o 3 specific IgG5 amounts during year 1 of the observation period, suggesting that other confounders or regional influences may have modified Cul o 3 specific IgG5 antibody measurements prior to the first *Culicoides* exposure in the former study by Ziegler et al. [7].

Clinical signs of allergy started to resolve at the end of *Culicoides* exposure and completely resolve during winter months in most horses. Simultaneously, significantly increased Cul o 3 specific IgG3/5 and Cul o 2 specific IgG5 were detected in allergic horses during late fall of year 2. This suggested that IgG antibodies provide a tool for detection of allergen specific immune responses beyond the actual exposure to *Culicoides* and distinguish allergic from non-allergic horses during the non-symptomatic phase that is currently lacking for CH. It is also noteworthy that IgG5 and IgG3 are the two IgG isotypes of the horse that have been associated with responses to extracellular pathogens and thus are most likely linked to T helper 2 and IL-4 mediated immune responses and precede IgE development in horses [44].

Surprisingly, allergen specific IgE levels in serum remained still very low during the first two years of *Culicoides* exposure when tested with our sensitive multiplex technique. What can be the reason for the relatively low IgE measurements in allergic horses? One reason could be that the total IgE concentration in serum is in the high ng/ml to low μg/ml range while most IgG isotypes occur in mg/ml concentrations [3]. Allergen specific IgE and IgG isotypes are also just a small but variable portion of the total serum antibody pool. Other studies have shown that allergen specific IgE levels increase with subsequent years of *Culicoides* exposure [7, 45] suggesting that circulating allergen-specific IgE amounts in serum may indeed still be quite low during the first and second year of *Culicoides* exposure. In addition, allergen specific IgG and IgE antibodies in serum compete for the binding to the allergen in the assay. Here, we have shown very high Cul o 2 and Cul o 3 IgG, and especially IgG5 and IgG3/5, amounts during the *Culicoides* exposure season. If serum is used as a sample, these IgG antibodies take up many allergen binding spaces in the assay. For example, if Cul o 2 coupled beads are representing the assay’s matrix, only a small proportion of the Cul o 2 allergen is available for Cul o 2 specific IgE binding corresponding to the relative portions of Cul o 2 specific IgE and IgG isotypes in the sample. As higher the IgE proportion as more likely it is that the IgE that bound to the Cul o 2 bead reaches the limit of detection of the IgE detection assay (few ng/ml Cul o 2 specific IgE). This happened in a few horses, as indicated by the large error bars in the IgE figures in the article, but not consistently in the whole group. However, allergen specific IgG in serum will always by far exceed allergen specific IgE amounts. Competition of binding site in serological assays is a general problem of allergy diagnostics and has been recognized previously [2]. It is a major weakness of allergen specific IgE detection in serum samples. Allergen specific IgG isotype measurements using allergen-isotype combinations that can distinguish allergic from non-allergic horses could help to improve serological allergy diagnostics.

## Conclusions

Out of nine allergens tested in this study we identified two, Cul o 2 and Cul o 3, that shared cross-reactive epitopes with the *Culicoides* species in the Northeast US and were valuable diagnostic tools to distinguish allergic and non-allergic horses. Based on previous findings from other groups in Europe and our US data shown here, Cul o 2 and Cul o 3 are likely major *Culicoides* allergens. These two allergens, in combination with IgG3/5 and IgG5 isotype evaluation, can be used to confirm the clinical diagnosis of CH and the allergen(s) responsible for clinical disease. In addition, we found the first evidence for a novel predictive biomarker for CH, Cul o 2 specific IgG3/5 that identified horses prior to the development of clinical allergy after they were exposed to *Culicoides*.

## Methods

### Horses and natural allergy model

Fifteen Icelandic mares and one stallion were purchased in Iceland from private owners. In February 2012, all 15 mares were pregnant and exported together with the stallion from Iceland to the US. At the time of import to the US, the mares were 5 to 13 years of age (median 8 years) and the stallion was 16 years old. Prior to the export date of the horses *Culicoides* had never been detected in Iceland and CH was never reported in Iceland. The immune system of the horses was thus considered naïve to *Culicoides* exposure by the time they entered the US. After arriving in the US, the horses were directly transported to Cornell University, where importation quarantine was performed under USDA-APHIS supervision as previously described [46].

Afterwards, the horses were released to a pasture area at Cornell University and were kept there as a herd without contact to other horses for the following two years. During the summer months, all horses were naturally exposed to *Culicoides* which are endemic in the environment from mid-May to mid-October during the first and second summer of the study period. This was confirmed by collecting *Culicoides* from the environment of the horses as previously described [6]. The wing pattern of *Culicoides* at Cornell University did not match any of the pattern reported earlier [47]. Horses lived outside 24/7 all year long with free access to running sheds, water and mineral salt blocks. They were all on the same diet composed of grass hay ad libitum during the winter months and free pasture grazing during the summer.

All mares foaled in June of the first summer (2012), were afterwards covered by natural breeding by the same stallion, and all 15 mares foaled again in June/July of the second summer (2013). In both years, the mares foaled without any complications and all foals were healthy. All adult horses were simultaneously vaccinated and dewormed as a group. Vaccinations were given against Rabies, Tetanus, West Nile virus, Eastern and Western Encephalitis virus in both years and against equine herpesvirus in the year 2. Deworming was performed in December 2011 prior to exportation from Iceland and then at Cornell University in March, August and December of 2012 and 2013. The horses did not receive any other treatments.

### Clinical scoring, allergy group assignment, and environmental recording

A clinical allergy scoring system was previously described and validated [23] and was used throughout this project to evaluate pruritus, alopecia, and dermatitis (Table 1). A clinical score was obtained from all horses 3–5 times per month and resulted in an average monthly score for each individual horse. To be assigned to the allergic group a horse had to have at least one monthly clinical score of > 3 including a skin irritation/dermatitis score of 1 or higher.

**Table 1:** Clinical scoring system for seasonal *Culicoides hypersensitivity* in horses used for the longitudinal evaluation of clinical signs of allergy

Clinical sign	Grade	Score	Maximal score
**Pruritus**	No mane or tail scratching	0	
	Mild mane and/or tail scratching	1	
	Moderate mane and/or tail scratching	2	
	Intense mane and tail scratching	3	3
**Alopecia**	None	0	
	Few broken hairs one location	1	
	Several locations with broken hairs	2	
	Moderate hair loss, mane or tail	3	
	Severe hair loss, mane and tail	4	4
**Skin irritation/dermatitis**	No skin irritation	0	
	Mild dermatitis, one location	1	
	Moderate dermatitis, several locations	2	
	Dermatitis with skin lesions	3	3
	**Total score**		10

Daily maximal (high) and minimal (low) outside temperatures were recorded throughout the study to determine if any environmental temperature differences occurred in the two years of the study that could have influenced a variation of *Culicoides* activity. The daily temperatures were used to calculate the average monthly high and low temperatures. The average monthly temperatures were then compared between years 1 and 2 to provide justification that the occurrence of clinical signs of CH in year 2 was not influenced by environmental temperature differences between the two years of the study period.

### Blood sampling

Blood samples were collected from the jugular vein with a vacutainer collection system without coagulant and an 18-gauge needle. Blood samples were allowed to clot for serum collection and were then centrifuged at 700 x *g* for 10 min. The serum from each sample was harvested, frozen, and stored at − 20 °C until the analysis for *Culicoides* specific antibodies was performed. Serum samples collected at seven different time points were analysed for this project (Fig. 1). Samples were taken: 1) prior to importation to the US while the horses were still in Iceland; 2) post importation to the US and prior to the 1st *Culicoides* exposure (March, year 1); 3) during the 1st *Culicoides* exposure (August, year 1); 4) in fall (November, year 1) and 5) winter after the 1st *Culicoides* exposure (February, year 2); 6) during the 2nd *Culicoides* exposure (July, year 2); and 7) in fall post 2nd *Culicoides* exposure (December, year 2). All animal procedures were approved by the Cornell University Institutional Animal Care and Use Committee (protocol #2011–0011).

### Allergens

Nine potential *Culicoides* allergens were included for antibody testing in serum (Table 2). Out of several reported potential allergens, the selection of allergens for antibody testing was made based on information from these reports on their ability to distinguish serological responses in allergic and non-allergic horses [6–9, 19, 21] and the expression of sufficient allergen amounts as soluble recombinant proteins. *C. sonorensis* allergens Cul s 1 (AY603565) and Cul s D7 (AY603569) were expressed in *E. coli* as previously described for other proteins [48]. *C. nubeculosus* allergens Cul n 3 (HM145952) and Cul n 4 (HM145952) were expressed in transgenic barley [49], and Cul n 8 (HM145956) in Pichia pastoris. *C. obsoletus* allergens Cul o 2 (KC339672) and Cul o 3 (KC339673) were also expressed in Pichia pastoris. Cul o 3 as well as Cul o 2 sequences each comprised two potential asparagine (N)-glycosylation sites and were detected to be glycosylated by Pichia. The native Cul o 3 and Cul o 2 variants represented the protein derived from the codon-optimized gene sequences with Pichia specific N-glycosylation pattern, while enzymatic deglycosylation delivered a variant of Cul o 3 (Cul o 3 degly) without the glycans attached. In addition, *C. obsoletus* apyrase (MN123717) was expressed in insect cells (sf+).

**Table 2:** *Culicoides* allergens used for bead coupling to provide the allergen specific matrix of the allergen multiplex assay

***Culicoides*** Species	Allergen	Fluorescent bead No.	Origin	Expression system
*C. obsoletus*	Cul o 2	40	Germany	Pichia pastoris
	Cul o 3 ^a^	39	Germany	Pichia pastoris
	Cul o 3 (degly) ^a^	37	Germany	Pichia pastoris
	Apyrase	41	Germany	Insect cells (sf+)
*C. nubeculosus*	Cul n 3	36	Iceland	Barley
	Cul n 4	34	Iceland	Barley
	Cul n 8	42	Germany	Pichia pastoris
*C. sonorensis*	Cul s 1	33	Cornell	*E. coli*
	Cul s D7	38	Cornell	*E. coli*

^a^ Cul o 3 was used as originally expressed in Pichia pastoris. The same protein was also used in a deglycosylated form

### Coupling of allergens to fluorescent-beads

Fluorescent color-coded microsphere beads (Luminex Corp., Austin, TX) were coupled with nine potential *Culicoides* allergens as outlined in Table 2. A total of 5 × 10^6^ beads were coupled with 100 μg of the respective allergen using a bead coupling procedure previously described in detail [50].

### *Culicoides* allergen multiplex assay

Allergen specific Ig isotypes in horse serum were quantified using a Luminex system (Luminex Corp., Austin, TX). Each assay was multiplexed with nine different color-coded beads coupled with an individual *Culicoides* allergen (Table 2) as described above. For isotype measurement, IgE and six IgG isotypes were detected separately by preparing multiple copies of the sample plates. The following biotinylated monoclonal anti-equine isotype specific antibodies were used for detection: anti-IgE clone 134 [51], anti-IgG1 clone CVS45 [52], anti-IgG1/3 clone 159 [53], anti-IgG4/7 clone CVS39 [52], anti-IgG5 clone 416 [53], anti-IgG3/5 clone 586 [53], and anti-IgG6 clone 267 [53].

For the assay run, Millipore Multiscreen plates (Millipore, Danvers, MA) were soaked with PBS-T (0.05% Tween20 in PBS) for 5 min. Then, the buffer was aspirated using an EL X 50 plate washer (Biotek Instruments Inc., Winooski, VT). Undiluted serum samples were applied to the wells followed by bead mix solution with a total of 5 × 10^3^ beads per allergen and microtiter well in PBN buffer (1% BSA and 0.05% sodium azide in PBS, pH 7.4). The plates were covered with a foil to be protected from light, incubated for 30 min at room temperature on a shaker, and washed afterwards. Then, the different biotinylated anti-isotype detection antibodies were added to the plates and incubated for 30 min on the shaker at room temperature. All detection antibodies were diluted 1:500 in PBN. After incubation, plates were washed again. Streptavidin-phycoerythrin (Invitrogen, Carlsbad, CA) diluted 1:100 in PBN was added to the plates, incubated for 30 min on the shaker at room temperature, and then washed. Beads were re-suspended in 100 μl of PBN and incubated for 15 min on the shaker at room temperature. Finally, the assay was analysed in a Luminex 200 instrument (Luminex Corp., Austin, TX).

### Statistical analysis

The average monthly clinical scores during the two-year time period were compared between allergic and non-allergic groups by repeated-measures ANOVA using Sidak multiple comparison tests and using GraphPad Prism 6 for Mac OS X, version 6 f. The average monthly low and high environmental temperatures were normally distributed (D’Agostino & Pearson normality tests) and were compared by paired t-tests. The IgE and IgG allergen multiplex result analyses were conducted in R software (R Project for Statistical Computing [http://www.r-project.org/]). *P* values of 0.05 were considered statistically significant. The clinical presentation of allergy (present/absent) was determined in the second summer and horses were classified as clinically allergic or non-allergic. Seven isotype antibodies were used to measure serum antibodies against each of nine potential *Culicoides* allergens, producing 63 (7 × 9) isotype-allergen variables [Additional file 1]. Statistical analyses were conducted on subsets of data corresponding to the four study questions. For question 1 we used a subset of data from August and November of year 1, and February of year 2, while for question 2 we used data from samples taken in October prior to importation and March of year 1. For questions 3 and 4, we used a subset of data acquired in July and December of year 2, respectively. Statistical analysis started with the screening of the individual 63 isotype-allergen variables to identify those that were able to differentiate among allergic and non-allergic horses. The screening involved (i) visual examination of boxplots and (ii) statistical testing using a Mann-Whitney test, which was selected due to a relatively small sample size and the non-parametric nature of the test. Isotype-allergen variables with Mann-Whitney *p*-values < 0.05 were selected for further univariable testing using logistic regression [Additional file 1]. In testing the hypotheses underlying questions 1 and 2, the interest was in evaluating the combined effect of date and isotype-allergen variable using an interaction term. The effect of date alone was tested but was not significant. In the hypothesis testing as part of questions, 3 and 4 multivariable models were developed but none improved the model fit. Likewise, multivariable models with more than one isotype-allergen variable were tested but none improved the model fit. The model fit was evaluated based on the Likelihood Ratio test and Akaike’s Information Criterion.

## Supplementary information

**Additional file 1.** Results of Mann-Whitney test in initial analysis of all data points. Significant combinations from four questions asked are highlighted and were further analyzed by logistic regression.

**Additional file 2. **Cul o 2 specific antibodies in serum of allergic and non-allergic horses. Antibodies in serum of allergic (*n* = 9) and non-allergic (*n* = 7) horses were determined using a *Culicoides* allergen multiplex assay. (A) Cul o 2 specific IgG1, (B) IgG1/3, (C) IgG4/7, (D) IgG6, and (E) IgE. Horses were imported to the US in the beginning of year 1 (arrow). The dotted lines represent the natural exposure times to *Culicoides* midges during the two-year study period. MFI = median fluorescence intensity.

**Additional file 3. **Cul o 3 specific antibodies responses in serum of allergic and non-allergic horses. Longitudinal data representing (A) Cul o 3 specific IgG1, (B) IgG1/3 (C) IgG4/7 (D) IgG5, (E) IgG6, and (F) IgE antibodies in serum of allergic (n = 9) and non-allergic (n = 7) horses during the two-year study period. The arrow shows the time of import of the horses to the US. The dotted lines indicate natural exposure to *Culicoides* midges. MFI = median fluorescence intensity.

**Additional file 4.** Original data file.

## Data Availability

The datasets used and/or analyzed during the current study are available from the corresponding author on reasonable request.

## Abbreviations

CH: *Culicoides* hypersensitivity
Ig: Immunoglobulin
mAb: Monoclonal antibody
IgE: Immunoglobulin E
IgG: Immunoglobulin G
IgG1: Immunoglobulin G1
IgG1/3: Immunoglobulin G1/3
IgG3/5: Immunoglobulin G3/5
IgG4/7: Immunoglobulin G4/7
IgG5: Immunoglobulin G5
IgG6: Immunoglobulin G6
*C. nubeculosus*: *Culicoides nubeculosus*
*C. obsoletus*: *Culicoides obsoletus*
*C. sonorensis*: *Culicoides sonorensis*
Cul n 3: *Culicoides nubeculosus* allergen 3
Cul n 4: *Culicoides nubeculosus* allergen 4
Cul n 8: *Culicoides nubeculosus* allergen 8
Cul o 2: *Culicoides obsoletus* allergen 2
Cul o 3: *Culicoides obsoletus* allergen 3
Cul s 1: *Culicoides sonorensis* allergen 1
Cul s D7: *Culicoides sonorensis* allergen D7
PBS-T: Phosphate buffer saline with Tween20
PBS: Phosphate buffer saline
PBN: Phosphate buffer saline with bovine serum albumin and sodium azide
BSA: Bovine serum albumin
ANOVA: Analysis of variance
