# Supplementary material for: Cul o 2 specific IgG3/5 antibodies predicted Culicoides hypersensitivity in a group imported Icelandic horses
Source: BMC Vet Res. 2020 Aug 10;16:283. doi: 10.1186/s12917-020-02499-w (PMC7418374; doi:10.1186/s12917-020-02499-w)
Supplement: Supplementary file 1 — Additional file 1. Results of Mann-Whitney test in initial analysis of all data points. Significant combinations from four questions asked are highlighted and were further analyzed by logistic regression. [file 12917_2020_2499_MOESM1_ESM.docx]

| **Allergen-Ig combinations** | **Summer of year 2** | | **Late fall of year 2** | **Before *Culicoides* exposure** | **After *Culicoides* exposure in year 1 and before summer of year 2** |
| --- | --- | --- | --- | --- | --- |
| IgE_maltase | | 0.448750749 | 0.895308327 | 0.724547691 | 0.964213352 |
| IgE_Culn4 | | 0.1389248 | 0.775967284 | 0.371977616 | 0.607601739 |
| IgE_Culn3 | | 1 | 0.746646577 | 0.817964686 | 0.878675478 |
| IgE_Culo3.degly | | 0.219845973 | 0.039478315 | 0.634379699 | 0.615418053 |
| IgE_Culo3 | | 0.365444582 | 0.602729037 | 0.338207816 | 0.889021106 |
| IgE_Culo2 | | 0.789780556 | 0.36037387 | 0.851170408 | 0.516534813 |
| IgE_apyrase | | 0.710618677 | 0.632836911 | 0.991691916 | 0.819038058 |
| IgE_Culn8 | | 0.745333067 | 1 | 0.234435802 | 0.839690313 |
| IgE_D7 | | 0.827524386 | 0.115656799 | 0.914058277 | 0.035704818 |
| IgG1.3_maltase | | 0.837062937 | 0.915638572 | 0.486220701 | 0.648353073 |
| IgG1.3_Culn4 | | 1 | 0.041783217 | 0.386204877 | 0.561122694 |
| IgG1.3_Culn3 | | 0.757692308 | 0.837062937 | 0.421190735 | 0.925434927 |
| IgG1.3_Culo3.degly | | 0.407867133 | 0.873752613 | 0.843478574 | 0.732392729 |
| IgG1.3_Culo3 | | 0.710822257 | 0.469755245 | 0.119047554 | 0.371934912 |
| IgG1.3_Culo2 | | 1 | 0.071153846 | 0.757457754 | 0.37722494 |
| IgG1.3_apyrase | | 0.351048951 | 0.606468531 | 0.070338006 | 0.357101428 |
| IgG1.3_Culn8 | | 0.407867133 | 0.252272727 | 0.342362876 | 0.414047121 |
| IgG1.3_D7 | | 0.606468531 | 0.090734266 | 0.052673325 | 0.017920802 |
| IgG4/7_maltase | | 0.489831701 | 0.489187377 | 0.417512903 | 0.313524951 |
| IgG4/7_Culn4 | | 0.536013986 | 0.016433566 | 0.006682134 | 0.060013523 |
| IgG4/7_Culn3 | | 0.536013986 | 0.710822257 | 0.56061372 | 0.159638847 |
| IgG4/7_Culo3.degly | | 0.606468531 | 0.21048951 | 0.008825083 | 0.114787969 |
| IgG4/7_Culo3 | | 1 | 0.090734266 | 0.293884539 | 0.954498374 |
| IgG4/7_Culo2 | | 0.114160839 | 0.114160839 | 0.665395293 | 0.732416317 |
| IgG4/7_apyrase | | 0.837062937 | 0.4269227 | 0.270674462 | 0.189970378 |
| IgG4/7_Culn8 | | 0.407867133 | 0.299125874 | 0.933744087 | 0.924313256 |
| IgG4/7_D7 | | 0.873752613 | 0.560155681 | 0.270674462 | 0.270583474 |
| IgG1_maltase | | 0.791145929 | 0.263907519 | 0.23595486 | 1 |
| IgG1_Culn4 | | 0.351048951 | 0.022902098 | 0.051988243 | 0.750222058 |
| IgG1_Culn3 | | 0.680594406 | 0.918181818 | 0.836911457 | 0.536044588 |
| IgG1_Culo3.degly | | 0.299125874 | 1 | 0.454341986 | 0.493569596 |
| IgG1_Culo3 | | 0.873752613 | 0.407867133 | 0.81106491 | 0.675996795 |
| IgG1_Culo2 | | 1 | 0.022902098 | 0.79502557 | 0.87921741 |
| IgG1_apyrase | | 0.21048951 | 0.918181818 | 0.677639078 | 0.721959118 |
| IgG1_Culn8 | | 0.21048951 | 0.071153846 | 0.382720175 | 0.266600808 |
| IgG1_D7 | | 1 | 0.918181818 | 0.240294126 | 0.010074572 |
| IgG5_maltase | | 0.12426763 | 0.536013986 | 0.037638181 | 0.35144569 |
| IgG5_Culn4 | | 0.351048951 | 0.606468531 | 0.788988412 | 0.750222058 |
| IgG5_Culn3 | | 0.173776224 | 0.351048951 | 0.804885342 | 0.287451241 |
| IgG5_Culo3.degly | | 0.011538462 | 0.022902098 | 0.216235024 | 0.177288205 |
| IgG5_Culo3 | | 0.114160839 | 0.252272727 | 0.539718878 | 0.23129684 |
| IgG5_Culo2 | | 0.407867133 | 0.022902098 | 0.214588993 | 0.470407322 |
| IgG5_apyrase | | 0.173776224 | 0.918181818 | 0.820861786 | 0.156317809 |
| IgG5_Culn8 | | 0.173776224 | 0.173776224 | 0.102788488 | 0.834429876 |
| IgG5_D7 | | 0.21048951 | 0.606468531 | 0.298729859 | 0.834489712 |
| IgG3.5_maltase | | 0.536013986 | 0.710822257 | 0.003618327 | 0.077303481 |
| IgG3.5_Culn4 | | 0.351048951 | 0.606468531 | 0.496373499 | 0.318870293 |
| IgG3.5_Culn3 | | 0.114160839 | 0.252272727 | 0.19231133 | 0.750222058 |
| IgG3.5_Culo3.degly | | 0.000699301 | 0.007867133 | 0.073699042 | 0.511528128 |
| IgG3.5_Culo3 | | 0.022902098 | 0.536013986 | 0.473349071 | 0.183589512 |
| IgG3.5_Culo2 | | 0.21048951 | 0.002097902 | 0.02813977 | 0.077141756 |
| IgG3.5_apyrase | | 0.837062937 | 0.299125874 | 0.342362876 | 0.86620766 |
| IgG3.5_Culn8 | | 0.299125874 | 0.837062937 | 0.124861931 | 0.128608113 |
| IgG3.5_D7 | | 0.606468531 | 0.536013986 | 0.05810578 | 0.049436326 |
| IgG6_maltase | | 0.750111916 | 1 | 0.12722905 | 0.038166842 |
| IgG6_Culn4 | | 0.790542389 | 0.957509316 | 0.875988581 | 0.457940899 |
| IgG6_Culn3 | | 0.266028098 | 1 | 0.983579255 | 0.879206423 |
| IgG6_Culo3.degly | | 0.829601684 | 0.513019796 | 0.087368393 | 0.055876107 |
| IgG6_Culo3 | | 0.831359055 | 1 | 0.484782639 | 0.689183667 |
| IgG6_Culo2 | | 0.112074586 | 0.12426763 | 0.048292992 | 0.183549331 |
| IgG6_apyrase | | 0.837062937 | 0.536013986 | 0.146908235 | 0.115867329 |
| IgG6_Culn8 | | 0.469755245 | 0.595271383 | 0.289156883 | 0.494084965 |
| IgG6_D7 | | 0.173776224 | 0.837062937 | 0.836911457 | 0.924313256 |

**Additional file 1: Results of Mann-Whitney test in initial analysis of all data points.** Significant combinations from four questions asked are highlighted and were further analyzed by logistic regression.
