# Supplementary material for: Cul o 2 specific IgG3/5 antibodies predicted Culicoides hypersensitivity in a group imported Icelandic horses
Source: BMC Vet Res. 2020 Aug 10;16:283. doi: 10.1186/s12917-020-02499-w (PMC7418374; doi:10.1186/s12917-020-02499-w)
Supplement: Supplementary file 2 — Additional file 2. Cul o 2 specific antibodies in serum of allergic and non-allergic horses. Antibodies in serum of allergic (n = 9) and non-allergic (n = 7) horses were determined using a Culicoides allergen multiplex assay. (A) Cul o 2 specific IgG1, (B) IgG1/3, (C) IgG4/7, (D) IgG6, and (E) IgE. Horses were imported to the US in the beginning of year 1 (arrow). The dotted lines represent the natural exposure times to Culicoides midges during the two-year study period. MFI = median fluorescence intensity. [file 12917_2020_2499_MOESM2_ESM.docx]

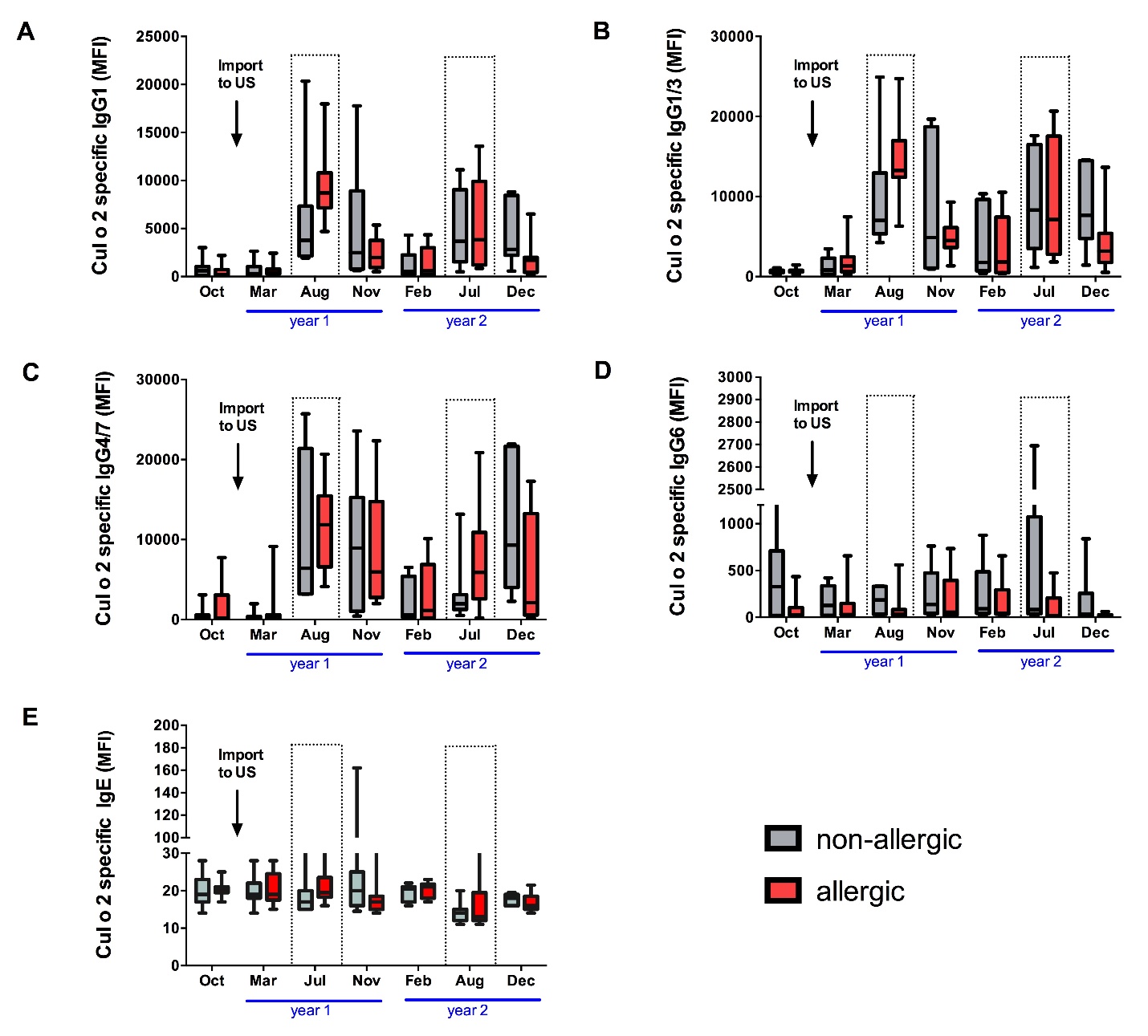


**Additional file 2: Cul o 2 specific antibodies in serum of allergic and non-allergic horses.** Antibodies in serum of allergic (n= 9) and non-allergic (n= 7) horses were determined using a *Culicoides* allergen multiplex assay. (A) Cul o 2 specific IgG1, (B) IgG1/3, (C) IgG4/7, (D) IgG6, and (E) IgE. Horses were imported to the US in the beginning of year 1 (arrow). The dotted lines represent the natural exposure times to *Culicoides* midges during the two-year study period. MFI = median fluorescence intensity.
