# Supplementary material for: Cul o 2 specific IgG3/5 antibodies predicted Culicoides hypersensitivity in a group imported Icelandic horses
Source: BMC Vet Res. 2020 Aug 10;16:283. doi: 10.1186/s12917-020-02499-w (PMC7418374; doi:10.1186/s12917-020-02499-w)
Supplement: Supplementary file 3 — Additional file 3. Cul o 3 specific antibodies responses in serum of allergic and non-allergic horses. Longitudinal data representing (A) Cul o 3 specific IgG1, (B) IgG1/3 (C) IgG4/7 (D) IgG5, (E) IgG6, and (F) IgE antibodies in serum of allergic (n = 9) and non-allergic (n = 7) horses during the two-year study period. The arrow shows the time of import of the horses to the US. The dotted lines indicate natural exposure to Culicoides midges. MFI = median fluorescence intensity. [file 12917_2020_2499_MOESM3_ESM.docx]

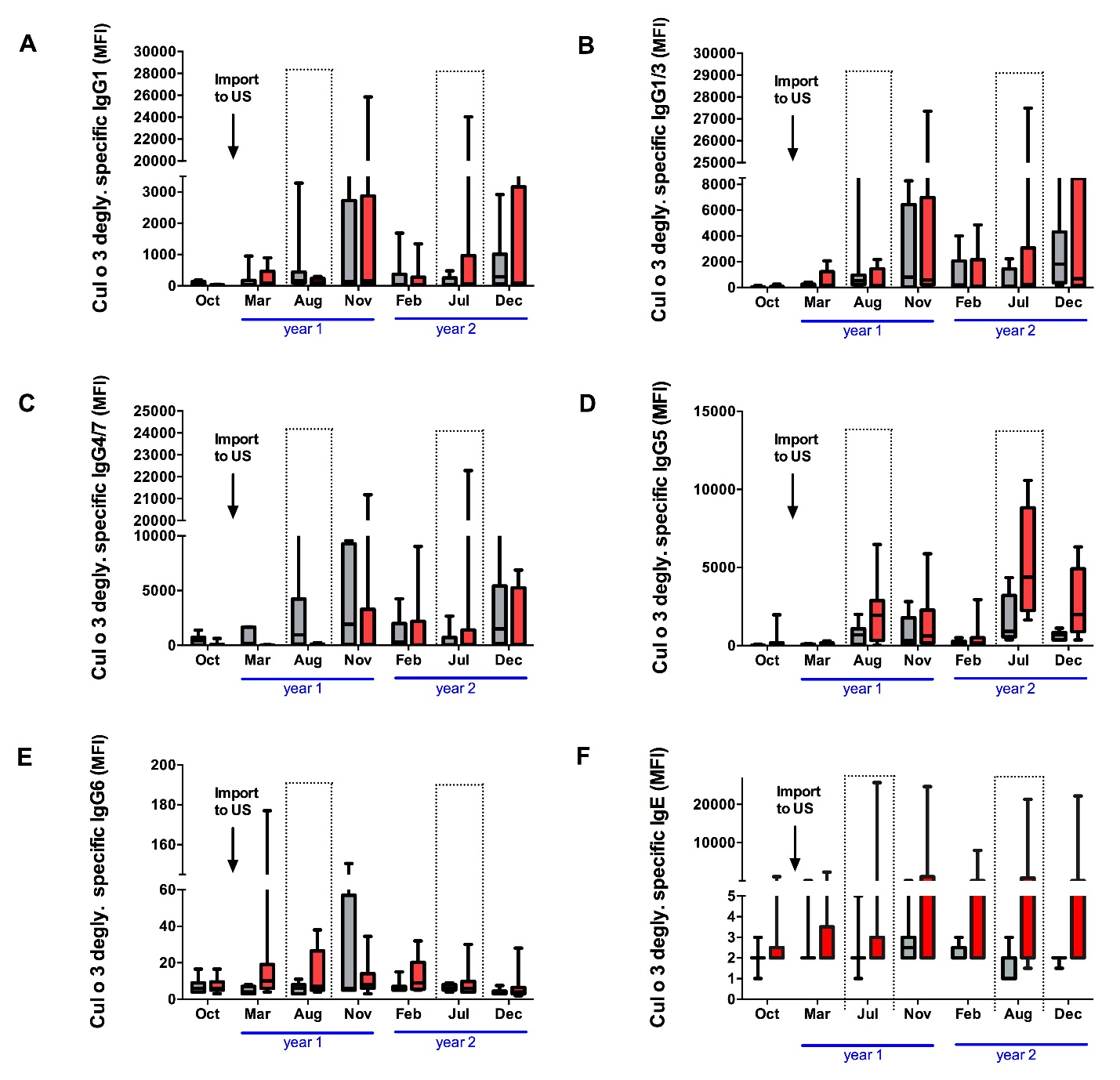

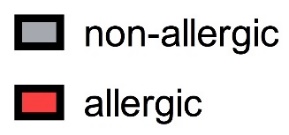


**Additional file 3: Cul o 3 specific antibodies responses in serum of allergic and non-allergic horses.** Longitudinal data representing (A) Cul o 3 specific IgG1, (B) IgG1/3 (C) IgG4/7 (D) IgG5, (E) IgG6, and (F) IgE antibodies in serum of allergic (n= 9) and non-allergic (n= 7) horses during the two-year study period. The arrow shows the time of import of the horses to the US. The dotted lines indicate natural exposure to *Culicoides* midges. MFI = median fluorescence intensity.
